# In-Vitro Safety Evaluation of Sodium Hypochlorite (NaOCl) as Part of Step 2 and Maintenance Therapy Protocols in Patients with Periodontitis Stages III-IV

**DOI:** 10.3290/j.ohpd.b4009557

**Published:** 2023-04-04

**Authors:** Giorgios Kardaras, Iasmina Marcovici, Darian Rusu, Cristina Dehelean, Dorina Coricovac, Vincenzo Iorio-Siciliano, Anton Sculean, Stefan-Ioan Stratul

**Affiliations:** a PhD Student, Department of Periodontology, Faculty of Dental Medicine, Anton Sculean Research Center for Periodontal and Peri-implant Diseases, Victor Babes University of Medicine and Pharmacy Timisoara, Romania. Performed the experiments in partial fulfillment of requirements for a PhD degree.; b Assistant Professor, Department of Toxicology, Drug Industry and Pharmaceutical Biotechnology, Faculty of Pharmacy, Research Center for Pharmaco-Toxicological Evaluations, Victor Babes University of Medicine and Pharmacy, Timisoara, Romania. Wrote the manuscript.; c Associate Professor, Department of Periodontology, Faculty of Dental Medicine, Anton Sculean Research Center for Periodontal and Peri-implant Diseases, Victor Babes University of Medicine and Pharmacy, Timisoara, Romania. Wrote the manuscript.; d Professor, Department of Toxicology, Drug Industry and Pharmaceutical Biotechnology, Faculty of Pharmacy, Research Center for Pharmaco-Toxicological Evaluations, Victor Babes University of Medicine and Pharmacy, Timisoara, Romania. Proofread the manuscript.; e Associate Professor Department of Toxicology, Drug Industry and Pharmaceutical Biotechnology, Faculty of Pharmacy, Research Center for Pharmaco-Toxicological Evaluations, Victor Babes University of Medicine and Pharmacy, Timisoara, Romania. Performed testing.; f Professor, Department of Periodontology, University of Naples Federico II, Naples, Italy. Proofread the manuscript.; g Professor, Department of Periodontology, University of Bern, Switzerland. Proofread the manuscript.; h Professor, Department of Periodontology, Faculty of Dental Medicine, Anton Sculean Research Center for Periodontal and Peri-implant Diseases, Victor Babes University of Medicine and Pharmacy Timisoara, Romania. Experimental design.

**Keywords:** periodontitis, primary gingival fibroblasts, sodium hypochlorite, toxicity

## Abstract

**Purpose:**

Since NaOCl acts as a strong oxidizing agent and presents potential toxicity, this study was adressed to evaluate the in-vitro safety of NaOCl solutions at concentrations below the limit of patient tolerance, i.e. ≥ 0.5%.

**Materials and Methods:**

First, an in-silico evaluation was conducted to predict the potential toxicity of NaOCl in terms of mutagenic, tumorigenic, irritant, and reproductive risks, as well as some drug-like properties of the molecule. The in-vitro experiments were based on 2D and 3D models. For the 2D approach, two selected cell lines – HaCaT (human skin keratinocytes) and HGF (human gingival fibroblasts) – were exposed to NaOCl at five concentrations (0.05 – 0.5%) for 10, 30, and 60 s to simulate possible clinical administration. The irritative potential of NaOCl 0.05% and 0.25% was assessed in a 3D in-vitro model (EpiDerm, reconstructed human epidermis). Statistical significance was set at p < 0.05.

**Results:**

The main findings suggest that NaOCl exerts cytotoxicity towards HaCaT immortalised keratinocytes and HGF primary gingival fibroblasts in a cell type-, dose- and time-dependent manner, with the most prominent effect being recorded in HaCaT cells after 60 s of treatment with NaOCl 0.5%. However, NaOCl was computationally predicted as free of mutagenic, tumorigenic, irritant, and reproductive toxicity, and showed no irritative potential in 3D reconstructed epidermis at concentrations of 0.05% and 0.25%.

**Conclusion:**

Further clinical and histological studies are required to confirm these results, as well as elucidate the potential cytotoxic mechanism induced by NaOCl in HaCaT and HGF cells at the tested concentrations.

Classified as one of most common inflammatory diseases^64^ with a global prevalence of up to 11%,^34^ periodontitis is a chronic disorder characterised by the accumulation of dental plaque (or biofilm) and progressive destruction of the tooth structures.^34,38^ Clinical features of periodontitis include gingival inflammation, bleeding, clinical attachment and alveolar bone loss, mobility, and pathologic migration.^34^ Aetiologically, periodontitis is multifactorial; as a disease of infectious origin,^20^ periodontitis is mainly caused by the development of bacterial biofilm on the tooth surface.^26,50^ Very recently, an overall concept suggested that this microbial biofilm can be considered a human tissue of bacteriological origin.^10,30^ Several patient-related factors such as genetics, inflammatory response, lifestyle habits (e.g. smoking), and systemic health could also influence its clinical presentation, progression, and outcome.^7,38^ A link has been found between periodontitis and serious systemic diseases, such as cancer, Alzheimer’s or cardiovacular disorders, thus increasing the importance of periodontal treatment.^56^

The treatment aims at preventing disease progression, minimising disease symptoms and perception, restoring lost tissues, and promoting periodontal self-care among patients. The therapeutic interventions, as revised by the latest EFP S3-level Clinical Practice Guidelines for Treatment of Stage I-III^49^ and Stage IV,^22^ include oral hygiene recommendations, smoking cessation support and dietary instructions, followed by plaque removal through subgingival instrumentation, adjunctive pharmacotherapy and surgery.^19,61^

The development of an effective and low-cost self-care method able to prevent and treat periodontal diseases has been considered a priority.^19^ One such affordable and effective option for the management of severe periodontitis is sodium hypochlorite (NaOCl), which can be applied as rinsing or irrigating solutions by patients during both step 1 (supragingival biofilm control) and 2 (subgingival instrumentation) of periodontal therapy as well as during periodontal maintenance to effectively remove and/or prevent dental plaque, gingival inflammation, and bleeding.^49,57^ Already in 1984, the American Dental Association (ADA) recommended NaOCl for use as a topical antimicrobial.^3^ NaOCl has many applications as an anti-microbial agent worldwide,^22^ and is one of the most commonly used canal irrigants in endodontics,^31^ owing to its rapid bactericidal action, broad-spectrum anti-microbial activity, and high efficacy in dissolving necrotic pulp residues and dentinal collagen.^5,13^ In endodontic irrigation, the living cells with which irrigants come into contact are mainly periodontal fibroblasts, endothelial cells and defense cells (plasma cells, macrophages, lymphocytes).^6^ NaOCl is employed at concentrations varying from 0.5% to 5.25%.^2^ For periodontal therapy, various concentrations of NaOCl solutions have been proposed, from 0.05% to 5.6%, depending on the procedure.^1,8,12,15,17,19,27,28,32,33,37,53-55,59^

The recommended duration of contact between NaOCl solutions and oral tissues also varies largely. It has been shown in vitro that 0.5% NaOCl was the lowest concentration able to eradicate bacteria within 15 s.^43^ In clinical studies, while rinsing sessions may last from 30 s–60 s,^12,15,17,19^ the irrigation time of gingival sulci and periodontal pockets depends on the amount of solution and the type of irrigation device employed.^8,17,33,37^ The activity of NaOCl is pH-dependent, with its strongest effect being achieved at neutral and slightly acidic pHs^31^ when it is found predominantly as hypochlorous acid (HOCl) – a highly reactive form of NaOCl responsible for its antibacterial and antifungal properties.^13,43^ HOCl acts by disrupting the cellular components (e.g. wall, membrane) and macromolecules (e.g. proteins, lipids) of microorganisms.^23^

Despite the benefits, NaOCl solutions have an unpleasant smell and taste, making them less suitable candidates for daily use as oral antimicrobials. Moreover, NaOCl is a strong oxidant, leading to potential toxicity and corrosive action if it contacts the skin or mucous membranes.^52,65^ Currently, the literature does not contain evaluations of the cytotoxic effects of NaOCl rinses used adjunctively as antimicrobials during steps 1 and 2 of periodontal therapy and during periodontal maintenance, when it contacts the most common oral cells – gingival fibroblasts and keratinocytes.

Based on the above considerations and using computational predictions as well as 2D and 3D in-vitro methods, the present study aimed to investigate the potential toxicity exerted by various concentrations of NaOCl (as recommended by recent protocols for the treatment of stage III-IV periodontitis [≤ 0.5%]) on primary gingival fibroblasts and human immortalised keratinocytes.

## Materials and Methods

### Reagents

The following reagents were used in the present experiments: NaOCl, commercially available as Chloraxid 2% (Cerkamed Medical Company; Poland); specific cell culture media (Dulbecco’s Modified Eagle’s Medium [DMEM], ATCC 30-2002; American Type Culture Collection, Manassas, VA, USA), Fibroblast Basal Medium (ATCC PCS-201-030; American Type Culture Collection), cell culture supplements including Fibroblast Growth Kit-Low Serum (ATCC PCS-201-041; American Type Culture Collection), an antibiotics mixture (penicillin/streptomycin [ATCC 30-2300], and penicillin/streptomycin/amphotericin B [ATCC PCS-999-002]; American Type Culture Collection), heat-inactivated fetal bovine serum (FBS, Sigma-Aldrich; St Louis, MO, USA), phosphate saline buffer (PBS, Sigma-Aldrich), trypsin-EDTA solution (Sigma-Aldrich), and MTT assay determination kit (MatTek; Bratislava, Slovakia). The EpiDerm tissues (EPI-212; Lot no. 36155), their specific assay media (EPI-100-NMM), Dulbecco’s phosphate buffered saline (DPBS), sodium dodecyl sulphate (SDS) 5%, and the MTT kit were provided by MatTek. The reagents applied were of analytical purity and approved for cell culture use.

### Cell Lines

The cell lines selected for the realisation of the present study were of oral or dermal origin, as follows: oral origin: primary gingival fibroblasts, HGF (PCS-201-018; American Type Culture Collection) as a frozen vial; dermal origin: human immortalised keratinocytes, HaCaT (CLS Cell Lines Service; Eppelheim, Germany).

### Cell Culture Growth

Cells were cultured according to the manufacturer’s recommendations. HaCaT cells were cultured in DMEM supplemented with FBS (final concentration 10%) and a mixture of antibiotics (penicillin/streptomycin, final concentration 1%). HGF cells were grown in Fibroblast Basal Medium supplemented with Fibroblast Growth Kit-Low Serum and penicillin/streptomycin/amphotericin B solution. The cells were maintained in culture under standard conditions, i.e. at 37°C and 5% CO_2_ in a humidified CO_2_ incubator.

### Computational Predictions

To predict the potential toxicity of NaOCl, the open-source programme OSIRIS Property Explorer (a computational tool estimating a molecule’s mutagenic, tumorigenic, irritant, and reproductive toxicity) was used as described in previous studies.^4,47^ Since the programme offers other relevant properties (molecular weight [MW], calculated LogP [cLogP], solubility, drug-likeness, and drug score), these are also presented in the present work. The canonical SMILES (simplified molecular-input line entry system) for NaOCl was obtained from https://pubchem.ncbi.nlm.nih.gov/.

### Cell Viability Assessment using MTT Assay

The MTT (3-(4,5-dimethylthiazol-2-yl)-2,5-diphenyltetrazolium bromide) assay is a standard method applied to quantify the viability of cells by measuring their metabolic activity.^17^ It was selected in this study to evaluate the impact of NaOCl impact on cell viability. Furthermore, this method has high sensitivity regarding the evaluation of the cytotoxicity induced by dental materials.^48^ The experimental protocol applied in the present study was established based on previous studies as well as manufacturer’s specifications and adapted to our laboratory conditions. In brief, the cells were cultured in 96-well plates (10^4^ cells/200 µl/well) and treated with different concentrations of NaOCl (0.05%, 0.1%, 0.2%, 0.25% and 0.5%) for different durations (10, 30, and 60 s). The concentrations of NaOCl were obtained by dilution in the specific culture media from the 2% stock solution. After the treatment period, the old culture medium was replaced with 100 µl of fresh medium and 10 µl of MTT reagent/well, followed by 3 h of incubation at 37°C. The solubilisation buffer (100 µl/well) was added after the incubation step and the plates were kept at room temperature for 30 min protected from light. The absorbances values were measured at two wavelenghts (570 and 630 nm) using Cytation 5 (BioTek Instruments; Winooski, VT, USA).

### Cell Morphology

Changes in cell morphology represent a reliable marker to identify the potential cytotoxicity induced by the test substance. The impact of NaOCl on cell morphology and confluence was monitored microscopically using Cytation 1 (BioTek Instruments). The cells were observed under bright field illumination and were photographed at the end of the treatment periods. The obtained images were analysed using Gen Microplate Data Collection and Analysis Software (BioTek Instruments).

### Skin Irritation Assay

The skin irritative potential of NaOCl was assessed using the EpiDerm (EPI-212) reconstructed human epidermal model. The in-vitro skin irritation test (EPI-212-SIT), a testing method validated by the OECD 439, was performed according to the manufacturer’s recommendations and by following the steps described by Pinzaru et al.^44^ Two NaOCl concentrations were selected for this test, 0.05% and 0.25%, with the former being the lowest recommended by the literature in periodontal therapy, and the latter the highest recommended by Slots et al’s^56^ comprehensive antimicrobial protocol from 2020. The NaOCl solutions were prepared in DPBS which was used as the negative control (NC). The positive control (PC) selected in this assay was sodium dodecyl sulphate (SDS) 1%. The tissue viability (%) was calculated according to the following formulae:^44^

**Figure:**
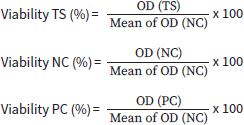


where TS = tested substance (NaOCl), NC = negative control (DPBS), PC = positive control (SDS 1%) and OD = optical density.

### Statistical Analysis

The results obtained were expressed as means ± SD. The difference between means was compared using one-way ANOVA, followed by Dunnett’s multiple comparison post-hoc test (GraphPad Prism version 6.0.0 for Windows, GraphPad Software; San Diego, CA, USA, www.graphpad.com). The difference between groups was considered statistically significant if p < 0.05.

## Results

### NaOCl Lacks Mutagenic, Tumorigenic, Irritant and Reproductive Toxicity According to Computational Predictions

To verify the safety of NaOCl, its potential toxicity was predicted using a computational analysis. The results (Table 1) indicate that NaOCl lacks mutagenic, tumorigenic, irritant and reproductive toxicity. Furthermore, it displays a negative Drug-likeness value (-1), but an overall positive Drug Score of 0.63. Some other properties of NaOCl (MW, cLogP, and solubility) are also presented.

**Table 1:** Computational prediction of the properties exerted by NaOCl, as generated by OSIRIS Property Explorer

MW	cLogP	Solubility	Drug-likeness	Drug Score	Toxicity			
					Mutagenic	Tumorigenic	Irritant	Reproductive
51	-4.16	0.25	-1	0.63	No risk	No risk	No risk	No risk

MW = molecular weight (g/mol); cLogP = calculated LogP.

### NaOCl Treatment Decreases HaCaT and HGF Cell Viability in a Dose- and Time-dependent Manner

**Fig 1:**
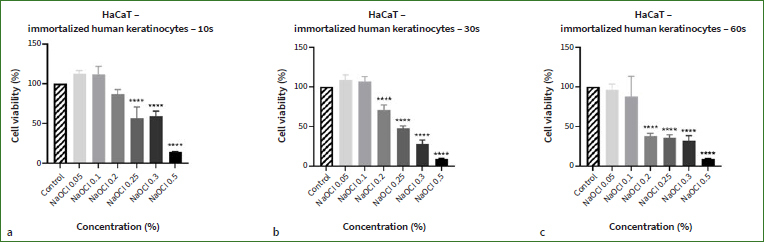
Assessment of NaOCl’s (0.05%, 0.1%, 0.2%, 0.25%, 0.3% and 0.5%) impact on human immortalised keratinocyte (HaCaT) viability after (a) 10, (b) 30, and (c) 60 s of treatment with MTT assay. The results are expressed as cell viability percentage (%) normalised to control (unstimulated) cells. The data represent the means ± SD of three independent experiments performed in triplicate. One-way ANOVA was applied to determine statistical differences vs control, followed by Dunnett’s multiple comparisons post-test (**** p < 0.0001).

The results indicated that the treatment of human skin keratinocytes (HaCaT with NaOCl for 10, 30, and 60 s) produced a dose-dependent decrease in cell viability (Fig 1). The highest concentration tested (0.5%) proved to be the most cytotoxic, reducing the cell viabilities to 14% (10 s), 9.5% (30 s) and 9.13% (60 s). The cytotoxicity of NaOCl was also time-dependent: at 10 s, statistically significant reductions in cell viability were noted starting with 0.25%, while at 30 s and 60 s no statistically significant viability decreases were detected up to 0.2%. A slight increase in HaCaT cell viability was detected when treated with NaOCl at low concentrations for 10 s (NaOCl 0.05%: 112.45%; NaOCl 0.1%: 111.6%) and 30 s (NaOCl 0.05%: 108.5%; NaOCl 0.1%: 107%). We also evaluated its effect on HaCaT cells after 24 h of incubation, at which point NaOCl was established as highly cytotoxic at all six concentrations (the percentage of viable cells was lower than 10%, data not shown).

**Fig 2:**
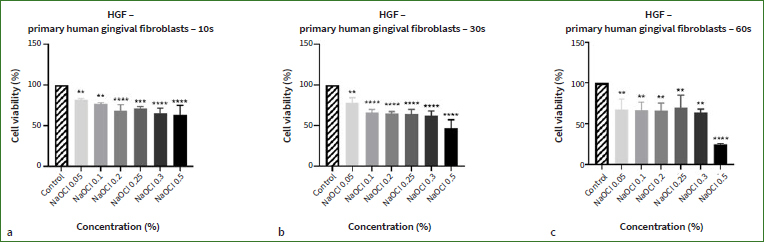
Assessment of NaOCl’s (0.05%, 0.1%, 0.2%, 0.25%, 0.3% and 0.5%) impact on primary human gingival fibroblast (HGF) viability after (a) 10, (b) 30, and (c) 60 s of treatment with the MTT assay. The results are expressed as cell viability percentage (%) normalised to control (unstimulated) cells. The data represent the means ± SD of three independent experiments performed in triplicate. One-way ANOVA was applied to determine statistical differences vs control, followed by Dunnett’s multiple comparisons test (** p < 0.01; *** p < 0.001; **** p < 0.0001).

A similar dose- and time-dependent response was noticed in the case of human primary fibroblasts (HGF) following NaOCl treatment for 10, 30, and 60 s (Fig 2). However, decreases in cell viability percentages were recorded at all concentrations and times. The highest cytotoxic effect was exerted by NaOCl 0.5%, which reduced the cell viability percentages to 63.5% (10 s), 46.9% (30 s), and 24.7% (60 s). Comparing the calculated IC50s (Table 2) for both cell lines at the three tested times shows that HaCaT cells are more sensitive to the toxicity of NaOCl compared to HGF (the IC50 values for HaCaT cells are lower than those for HGF cells).

**Table 2:** Calculated IC50 (%) values for NaOCl in HaCaT and HGF cells following 10, 30 and 60 s of treatment

Treatment time (s)	IC50 (%)	
	HaCaT	HGF
10	0.33	0.77
30	0.28	0.47
60	0.23	0.34

### NaOCl Treatment Induces Changes in HaCaT and HGF Cell Morphology

**Fig 3:**
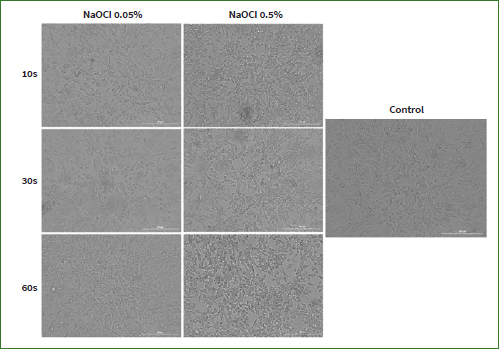
Morphological aspect and confluency of HaCaT cells following the three treatments (10, 30, and 60 s) with NaOCl 0.05% and 0.5% compared to control (untreated cells). The scale bars represent 200 µm.

**Fig 4:**
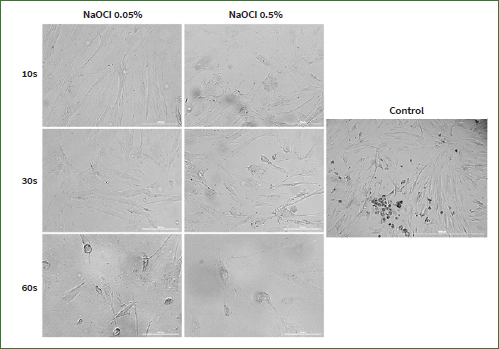
Morphological aspect and confluency of HGF cells following the three treatments (10, 30, and 60 s) with NaOCl 0.05% and 0.5% compared to control (untreated cells). The scale bars represent 200 µm.

Taking into account that morphological features represent key descriptors of cell death,^66^ another aspect evaluated in the present study was the effect induced by NaOCl on cell morphology. Based on the viability results (Figs 1 and 2), the lowest and the highest concentrations were selected for this evaluation (0.05%, and 0.5%). Normal morphology was evident in control (non-treated) cells. As shown in Fig 3, NaOCl 0.5% treatment for 10 s, 30 s, and 60 s induced visible changes in HaCaT cell shape and confluence: roundish cells, floating in the cell culture medium, and a lower confluence as compared to control cells (unstimulated cells). The same experimental protocol was applied for HGF cells. As shown in Fig 4, the most evident changes in cellular morphology and confluence were recorded following the 60 s treatment with NaOCl at both concentrations (0.05% and 0.5%) – the cells appear as round, floating, detached from the plate’s surface, and stressed compared to control – suggesting that the highest cytotoxicity of NaOCl was achieved after the longest treatment period (60 s). These data are in agreement with the results from the cell viability evaluation.

### NaOCl Lacks Irritative Potential in EpiDerm Skin Model Inserts

**Fig 5:**
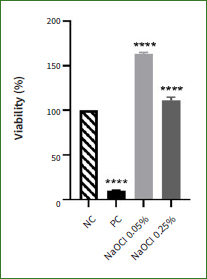
Viability percentage of EpiDerm skin model inserts (EPI-212-SIT) at 18 h post-treatment with negative control (NC; DPBS), positive control (PC; SDS 1%) and NaOCl (0.05% and 0.25%). One-way ANOVA and Dunett’s multiple comparisons test were applied to identify statistical differences between PC- and NaOCl-treated inserts and NC-treated inserts (****p < 0.0001).

The skin irritative potential of NaOCl (0.05%, and 0.25%) was assessed using 3D reconstructed epidermis inserts. The results (Fig 5) indicate that NaOCl exerts a stimulatory effect on the viability of the treated tissues at both tested concentrations compared to DPBS (positive control, PC): at 0.05% the viability significantly increased to 163%, while at 0.25% its value was 111.3%. SDS 1%, the chosen positive control (PC) for this assay, decreased tissue viability to 10.3% compared to NC.

## Discussion

The present paper evaluated NaOCl in vitro at doses which were recently recommended for the treatment of stage III-IV, grade C periodontitis (≤ 0.5%).^54,56^ The cytotoxicity of NaOCl solutions used as antimicrobials in oral treatments has been assessed in several studies.^52,65^ Previous data published in the literature mentioned that NaOCl doses recommended for endodontic irrigation (0.5-%5.25%) which proved to be both antimicrobial and capable of dissolving the remnants of necrotic tissue, are cytotoxic for the surrounding tissue.^2^ The most recent protocol for endodontic irrigation proposes lower doses for NaOCl, i.e. 0.1%-0.25%, which also proved to be effective.^57^ It is important to mention that in endodontic applications, the contact between oral tissues and irrigants is only accidental, being prevented by the use of the rubber-dam. In contrast, in periodontal application, both gingiva and oral mucosa are exposed to the irrigant/rinse, with occasional contact with the peri-oral tissues.

As an antimicrobial adjunctive in the treatment of inflammatory periodontal disease, high concetrantions (5.6%) were proposed decades ago to facilitate gingival curettage,^1,32^ while other authors proposed either subgingival irrigation with NaOCl solutions of 0.5%^8,33^ or oral rinses at concentrations varying between 0.05%^12^ and 0.1%,^15^ or both subgingival irrigations and rinses with 0.25% solutions.^17,19^ A NaOCl formulation as a gel containing 0.95% NaOCl was extensively used in recent years to treat periodontal^24,37^ and peri-implant^24^ infections, as well as during maintenance after periodontal therapy.^46^

A very recent protocol recommends 0.1%-0.25% sodium hypochlorite (freshly diluted Regular Clorox household bleach) oral rinses for 30 s twice weekly as patient self-care, for life-long use.^56^ This protocol combines betadine 10% subgingival irrigations with NaOCl rinses and lately drew particular interest in the treatment of severe forms of periodontitis, as it combines high antimicrobial efficiency with low-cost, affordable active substances. The authors of that protocol have modified the recommended concentrations (0.05%, 0.10%, 0.2%, 0.25%) several times over the last decades, motivated by the individual acceptability of the taste, smell and strength of the solutions by the patients.^27,28,53,54^ In general, the highest tolerated concentration limit for such NaOCl solutions has been set at 0.5%.^54^

However, to date, the potential toxicity of NaOCl at these concentrations lacks comprehensive verification. In the light of these data and taking into account the high frequency of using NaOCl as irrigant in dental medicine, the objective of the present study was to examine its potential toxicity in vitro at concentrations lower that 0.5%.

Human fibroblasts and keratinocytes are frequently used in the biological assessment of dental materials.^45,58^ For our research, HGFs were used for cytotoxicity testing, because in the oral cavity, they come in contact with substances used for dental biofilm control and with restorative dental materials, and are thus more clinically relevant. Also, HGFs are sensitive cells that can be easily isolated and cultured in normal culture medium.^19^ HaCaT cells are immortalised human skin keratinocytes that are suitable substitutes for oral keratinocytes because they can be easily grown and passaged indefinitely.^10,41^ They have been recognised as a promising tool for studying regulation of keratinisation in human cells.^57^ A variety of studies used HaCaT cells to investigate the progress of oral lichen planus.^61,62,66^

To ensure maximum benefit from the use of NaOCl solutions in disrupting periodontal biofilms and killing bacteria, its ideal antimicrobial concentrations must be correlated with its lowest toxicity. Based on the fact that several other studies used various concentrations of NaOCl in oral rinse formulations,^8,12,17,19,35^ a pilot test^15^ was conducted as part of a different study in 2017 and found a reduction of 66.6% in colony forming units per milliliter of saliva after rinsing with 0.1% NaOCl. Higher concentrations, although presenting larger inhibition halos, were found unpleasant by the volunteers in that study because of the strong smell and taste of bleach detected immediately after rinsing.^15^ However, the latter authors failed to find additional benefits of NaOCl solutions during subgingival instrumentation in reducing supragingival plaque, gingivitis, and/or microbial pathogens.

In the current paper, we found that: (i) the computational tool predicted NaOCl to be free of mutagenic, tumorigenic, irritation, and reproductive toxicity, and possess a positive Drug Score; (ii) NaOCl reduced the viability of HaCaT and HGF cells in a dose- and time-dependent fashion, with the most cytotoxic effect being recorded after 60 s of treatment with the highest concentration (0.5%); HaCaT cells were more sensitive to NaOCl, since the calculated IC50 values were lower compared to those estimated for HGF cells; and (iv) NaOCl lacked skin irritation potential, and significantly stimulated the viability of human reconstructed epidermal tissues at concentrations of 0.05% and 0.25%.

This study was initiated with an in-silico evaluation to predict the potential toxicity of NaOCl in terms of mutagenic, tumorigenic, irritant, and reproductive risks, as well as some drug-likeness properties of the molecule (Table 1). The computational assessment estimated that NaOCl presents no risk of the aforementioned toxic events, while possessing a positive Drug Score (0.63), which can be correlated with its current therapeutic applications in endodontics as a proteolytic and antimicrobial agent.^39^ NaOCl has been recored in the literature as lacking mutagenic, carcinogenic, teratogenic and allergic potential since it is naturally produced by some human cells (e.g. neutrophils, monocytes and macrophages).^11,23^ A recent experimental study also showed that NaOCl induced no DNA damage in murine fibroblast cells, suggesting a lack of genotoxic potential.^36^

The in-vitro experimental design employed both 2D and 3D models. In the 2D approach, two selected cell lines – HaCaT (human skin keratinocytes) and HGF (human gingival fibroblasts) – were exposed to NaOCl at five concentrations (0.05 – 0.5%) for 10, 30, and 60 s to simulate its possible clinical administration. HaCaT, an immortalised human skin epithelial cell line,^25^ is widely employed in scientific research,^13^ resembles isolated keratinocytes in terms of surface markers and functional activities,^9^ and possesses an advantageous proliferation rate.^13^ Human gingival fibroblasts (HGF) are also reliable in-vitro models due to their numerous advantages. They possess low susceptibility to microbial action, great regenerative characteristics, the ability to differentiate into different types of cells, including periodontal ligament cells, and diploid nature conferring an increased capability to provide evidence of the cytotoxicity of dental materials.^29^

Previous in-vitro studies explored the potential toxicity of NaOCl against healthy cells following an exposure time of minutes or hours. For instance, a study by Shi et al^51^ concluded that NaOCl exerted little cytotoxicity on L929 mouse fibroblasts at concentrations varying from 0.1 to 5% after 30 min of treatment, while strong cytotoxicity was recorded after 1 h (at concentrations >0.5%), 6 h and 12 h (at all tested concentrations) of treatment. NaOCl exerted high cytotoxicity against murine fibroblast cells (lineage 3T3-L1) at concentrations of 2.5% and 5.25%, while at the concentration of 1.25%, the cell viability reduction was not statistically significant.^36^ In a recent study which tested low concentrations (0.025% to 0.2%), NaOCl was highly cytotoxic against human gingival fibroblast (hGF) cells compared to other root canal irrigants (Ca(OCl)_2_, EDTA and chlorhexidine) upon 6 and 24 h of treatment.^29^ In another study, NaOCl 2.5% was shown to reduce the viability of mesenchymal stem cells to 34±1.94% after 48 h of incubation.^41^ Essner et al^15^ noted that NaOCl induces alterations in the morphology and number of immortalised human bone marrow mesenchymal stem cells (hTERT-MSC-C1) at concentrations of 0.16% and 0.33%. Massive cell toxicity (indicated by the presence of round and floating cells) was obtained following their treatment with NaOCl 0.33% for 10 and 15 min.^16^

Similar to these reports, our study indicates that the cytotoxic effect of NaOCl on human keratinocytes and fibroblasts – assessed via cell viability and morphological evaluations – was dependent on the tested concentration, exposure time, and cell line. 0.5% was the concentration that led to the highest reductions in cell viability (<15% for HaCaT cells; <65% for HGF cells) and the most detrimental changes in cell shape and confluence. Of the three selected treatments, 60 s of cell exposure to NaOCl proved the most critical to their viability and morphological aspect, and NaOCl exerted greater cytotoxicity on HaCaT cells than on HGF cells (HaCaT IC50 < HGF IC50). According to the data presented in the literature, the cytotoxicity of NaOCl confirmed in this study might be attributed to its high hydroxyl ion activity, which alters the cell membrane integrity by degrading its lipid structure and inactivating proteins.^2,42^

Finally, by acknowledging the importance of skin irritation tests in the evaluation of topical preparations,^42^ the irritative potential of NaOCl 0.05% and 0.25% was assessed in a 3D in-vitro system (EpiDerm, reconstructed human epidermis). NaOCl exerted a stimulatory effect on the viability of reconstructed epidermal inserts (up to 163% with NaOCl 0.05%, 111.28% with NaOCl 0.25%) when compared to the negative control DPBS (Fig 5). Since the tissue viability remained stable over the value of 50%, NaOCl can be classified as a non-irritant at the two tested concentrations, according to OECD Test Guideline 439. To the best of our knowledge, these are the first in-vitro results addressing the potential irritation effect of NaOCl in 3D-reconstructed epidermis.

## Conclusion

The computational tool predicted NaOCl to be free of severe toxic events. NaOCl proved to be non-irritative in a 3D in-vitro setting. However, in the 2D in-vitro experiments, NaOCl was cytotoxic against HaCaT keratinocytes and HGF fibroblasts, depending on cell type, tested concentration, and exposure time. To provide a complete analysis of the biocompatibility of NaOCl irrigation solutions (0.05%-0.5%) used in periodontitis treatment, further studies are required.
